# Paraneoplastic Neurological Syndromes and Beyond Emerging With the Introduction of Immune Checkpoint Inhibitor Cancer Immunotherapy

**DOI:** 10.3389/fneur.2021.642800

**Published:** 2021-04-09

**Authors:** Cristina Valencia-Sanchez, Anastasia Zekeridou

**Affiliations:** Departments of Neurology and Laboratory Medicine and Pathology, Mayo Clinic College of Medicine, Rochester, MN, United States

**Keywords:** cancer immunotherapy, autoimmune encephalitis, immune-related adverse events, myasthenia, myositis, paraneoplastic neurological syndromes

## Abstract

Paraneoplastic neurological syndromes are more commonly seen with malignancies such as small cell lung cancer, thymoma, gynecological malignancies, and breast cancer as well as seminoma. With the introduction of immune checkpoint inhibitor (ICI) cancer immunotherapy we see an increase of autoimmune neurological complications in patients with malignancies not traditionally associated with paraneoplastic neurological syndromes, such as melanoma and renal cell carcinoma. Immune checkpoint inhibitors enhance antitumor immune responses resulting often in immune-related adverse effects that can affect any organ, including the central and peripheral nervous system, neuromuscular junction and muscle. Neurological complications are rare; neuromuscular complications are more common than central nervous system ones but multifocal neurological presentations are often encountered. The vast majority of neurological complications appear within 3 months of ICI initiation, but have been described even after ICI cessation. Neural autoantibody testing reveals autoantibodies in approximately half of the patients with CNS complications. Early suspicion and diagnosis is critical to avoid worsening and improve outcomes. Therapeutic strategies depend on the severity of the symptoms and initially typically involve discontinuation of ICI and high dose steroids. Further immunosuppression might be necessary. Outcomes are dependent on patient's characteristics and clinical presentations.

## Introduction

The introduction of immune checkpoint inhibitors (ICI) has revolutionized the treatment of various malignancies. ICI are monoclonal antibodies that target negative regulatory steps in T-cell activation; the most commonly used are against the cytotoxic T lymphocyte associated antigen 4 (CTLA-4) (ipilimumab, tremelimumab), the programmed death-1 receptor (PD-1) (pembrolizumab, nivolumab, and cemiplimab) and its ligand PD-L1 (atezolizumab, avelumab, and durvalumab).

Since ipilimumab was approved in 2011 for the treatment of metastatic melanoma, multiple new agents have been approved and the tumor indications have expanded, including non-small cell lung carcinoma (NSCLC), renal cell carcinoma, advanced Hodgkin lymphoma, urothelial carcinoma, small-cell lung cancer (SCLC) and Merkel cell carcinoma, amongst others (1–20).

These novel cancer immunotherapies enhance antitumor immune responses by blocking signaling pathways that have inhibitory effects on T-cell activation. By enhancing endogenous immune responses, ICI may trigger immune-related adverse events (irAEs) that can affect any organ; these include neurological complications. The severity of the irAEs is graded by the Common Terminology Criteria for Adverse Events (CTCAE) according to clinical severity. Neurologic irAE that limit self-care activities are considered grade 3, if they are life-threatening and require urgent intervention grade 4, and grade 5 if fatal (21).

Although the reported rate of irAE with ICI is high (up to 90%), the prevalence of severe (grade ≥ 3) irAE is ~15–42% with CTLA4 ICI, 5–10% with PD-1 ICI, and 1–7% with PD-L1 ICI. The combination of treatment with both CTLA-4 and PD1/PD-L1 ICI agents is associated with a higher risk of irAE than monotherapy, and up to 40–45% are severe. Frequent irAEs include dermatitis, colitis, hepatitis, thyroiditis and hypophysitis. The majority of these are reversible (22). Neurological immune complications are of particular concern given that they can be disabling and life threatening. In clinical trials, the overall incidence of neurological adverse events was 3.8% with CTLA4 ICI, 6.1% with PD1 ICI, and 12% with the combination of both. Most neurologic irAEs were mild, such as headache, and peripheral neuropathy. The incidence of neurologic irAE grade 3 or 4 was <1% (23, 24). Subsequent studies have reported a frequency of neurological complications with ICI of ~2–4% (24–26), and 1.5% for grades 3 or 4 (27). There is also a higher incidence of neurological irAE in males, which may also reflect the differences in epidemiology of the cancers in which ICI are used (28, 29).

Neurological irAE usually develop within 3 months after initiation of ICI treatment (24, 27, 28, 30, 31), although they may have a delayed onset, in some cases months after discontinuation of ICI (21). The clinical manifestations may involve any level of the neuraxis, and the presentation is often multifocal. They may also co-occur with non-neurological irAEs (27). The most prevalent neurological irAE except hypophysitis are neuromuscular complications. The prevalence of central nervous system (CNS) autoimmune complications is <1% (28, 29).

A subset of neurological irAEs triggered by ICI are compatible with paraneoplastic neurological disorders such as limbic encephalitis or subacute cerebellar ataxia and are often accompanied by classic paraneoplastic antibodies (30, 32). Interestingly, ICI can trigger paraneoplastic neurological disorders even in patients with tumors that are not typically associated with spontaneous paraneoplastic syndromes, such as melanoma or renal cell carcinoma (30). Additionally, spontaneous paraneoplastic neurological syndromes typically precede cancer diagnosis, and if detected, the tumor is usually at a limited stage. Conversely, patients who develop paraneoplastic syndromes as a complication of treatment with ICI, typically have advanced stage cancer with metastasis (33).

The increasingly reported cases and series have allowed a more clear understanding of the spectrum of neurologic irAE associated with ICI. Here we review the pathogenic mechanisms, clinical presentation, diagnostic approach, and therapeutic management of these complications.

## ICI Mechanism of Action

**Figure 1:**
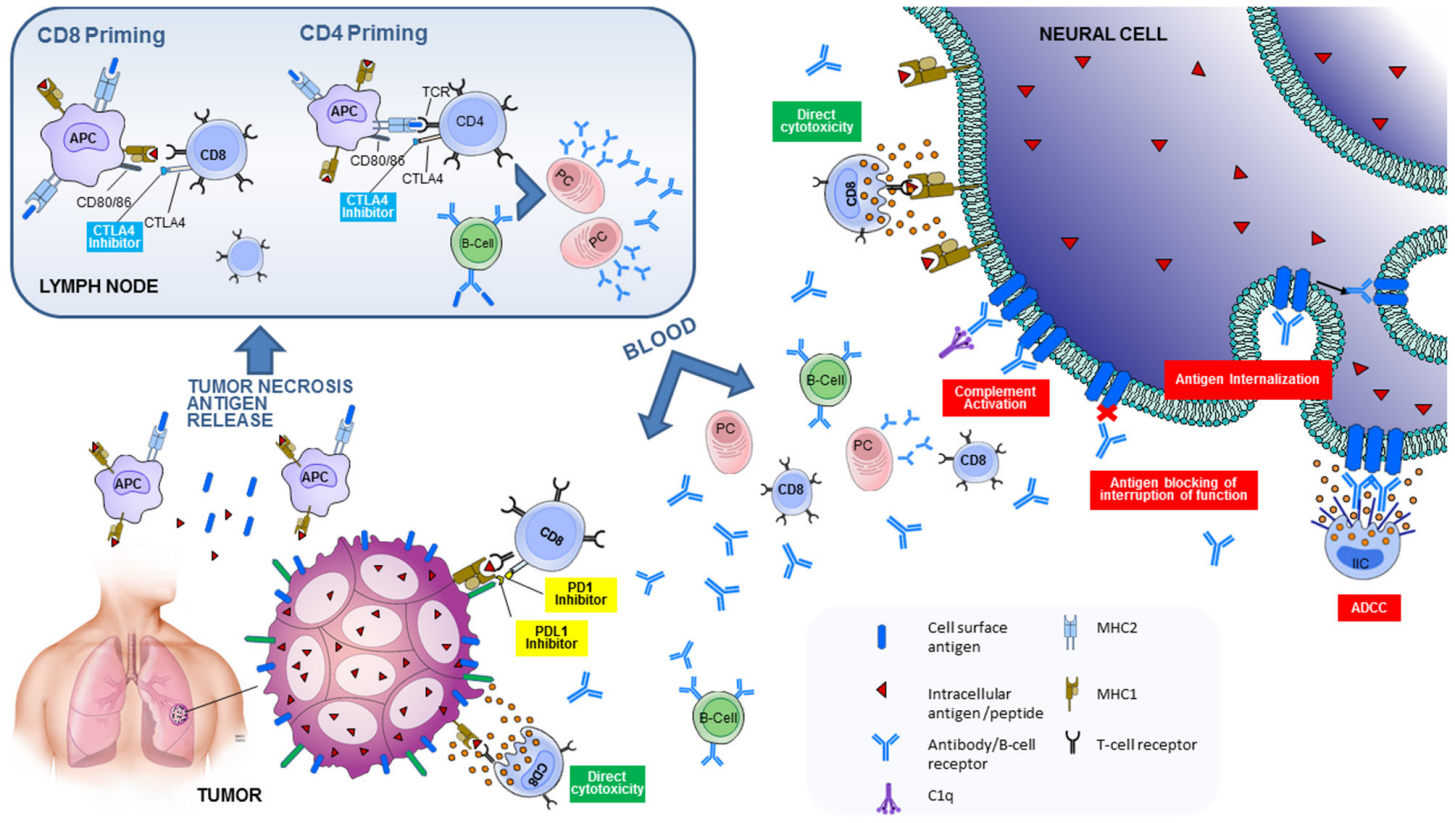
Pathogenic mechanisms involved in neurological immune related adverse events triggered by immune checkpoint inhibitors. Tumor antigens released from necrotic tumor cells are captured, processed into peptides by antigen-presenting cells (APC) that present them on major histocompatibility complex (MHC) molecules to the naïve T-cells in the lymph nodes. The T-cell receptor (TCR) engages with MHC molecules on the APC. Co-stimulatory signals from the interaction between CD28 on T-cells and B7 (CD80/86) on the APC are necessary to activate a naïve T-cell. This results in activation of CD8 cytotoxic T-cells against tumor-specific antigens, and CD4 helper T-cells that will help activate B-cells that have engaged via their B-cell receptor their cognate antigen and push them into antibody-producing plasma cells (PC). There are also inhibitory signals, or immune checkpoints, that regulate T-cell activation. CTLA4 on T-cells competes with CD28 for B7 binding. The interaction between CTLA4 and B7 is an inhibitory signal for T-cells. CTLA4 blockade therefore leads to enhanced T-cell activation in the lymph node. CD8 cytotoxic T-cells and plasma cells travel to the tumor. PDL1 expressed by the antigen-specific T-cells binds to PD-L1 expressed by tumor cells, leading to an inhibitory signal. Blockade of PD1/PD-L1 leads to enhanced T-cell activation on a tissue level. CD8 cytotoxic T-cells and plasma cells also travel to the nervous system. CD8 cytotoxic T-cells directed against neural antigens (that are aberrantly expressed by the tumor) will cause direct cytotoxicity and cell death. Antibodies directed against cell-surface neural antigens (aberrantly expressed by the tumor) can cause cell damage via several different pathways: by complement activation or antibody dependent cellular cytotoxicity (ADCC), by antigen internalization (modulation), or by antigen blocking with interruption of its function. Reproduced with permission from Sechi and Zekeridou (35). Suggested mechanisms of neurological autoimmunity in the context of ICI treatment.

Tumor antigens released from necrotic tumor cells are captured, processed into peptides by antigen-presenting cells (APC) that present them on major histocompatibility complex (MHC) molecules to the naïve T-cells in the lymph nodes. Except for the TCR engagement with MHC molecules on the APC, co-stimulatory pathways are necessary in order to activate a naïve T-cell. This results in immune activation against cancer-specific antigens via the primed CD8 cytotoxic T-cells but also through the CD4 helper T-cells that will help activate B-cells that have engaged via their B-cell receptor their cognate antigen and push them into antibody-producing cells (34). Activated effector T-cells infiltrate the tumor and bind to cancer cells that present their antigenic peptide bound to MHC-I on their cell surface leading to cell death. T-cell responses initiated by the interaction between TCR and MHC-presented antigenic peptides, are regulated by co-stimulatory and inhibitory signals. The inhibitory signals are the immune checkpoints, which are important in physiological conditions to prevent autoimmunity and regulate immune response both at the priming of the immune response and at a tissue level (Figure 1) (36). However, within the tumor microenvironment, increased expression of inhibitory molecules, amongst other factors, may dampen T-cell antitumor response (37). ICI are monoclonal antibodies that target these immune-inhibitory molecules, expressed by T-cells, such as CTLA-4 and PD-1, as well as the PD-1 ligand expressed by tumor cells but also epithelial cells and others in physiological conditions, resulting in enhanced immunity and often autoimmunity (38).

### CTLA-4 Pathway

CTLA-4 is expressed predominantly on activated T- cells and participates in downregulation of T-cell responses in the priming of the immune response at the lymph node. In resting T-cells, CTLA-4 is an intracellular protein. When the TCR engages with the antigen peptide-MHC on the APC, CD28 (on naïve T-cell) interaction with CD80/CD86 (on APC) is the second signal necessary for T-cell activation and amplification of TCR signaling. CTLA-4 then translocates to the cell surface, and competes with CD28 for binding to the costimulatory molecules CD80/CD86. CTLA4 molecules have higher affinity for binding than CD28 to the CD80/CD86 molecules and lead to arrest of T-cell activation and proliferation. In addition they might lead to sequestration of the CD80/CD86 molecules on the APC inhibiting further the CD28 costimulatory pathway. CTLA-4 is predominately found on CD4+ cells rather than CD8+ cells; its blockade leads to enhanced T cell activation on the lymph node level (39, 40).

### PD-1 and PD-L1 Pathway

PD-1 is a negative regulator of immune responses, which when engaged by its ligand (PDL1 or PDL2) limits effector T-cell responses in peripheral tissues (limiting inflammatory response to infection and autoimmunity) and tumors (limiting antitumor T-cell response). PD-1 is expressed on the surface of activated T-cells upon TCR engagement, and is highly expressed on tumor-infiltrating lymphocytes (39).

PD-1 has two ligands, PD-L1 and PD-L2 that are expressed by many somatic cells upon exposure to proinflammatory cytokines (39, 40). Tumor cells also may express on their surface PD-L1 and PD-L2 (41). The interaction between PD-L1 on tumors with PD1 on tumor infiltrating T-cells leads to downregulation of effector T-cell responses (42). Monoclonal antibodies against PD-1 and PD-L1 block this interaction. PD-1/PDL1 pathway blockade results in preferential stimulation of antitumor T-cells (40). PD-1 is also expressed in other immune cells such as natural killer (NK) cells and B-cells. Therefore, PD-1 blockade may also enhance NK cell activity in tumors, and antibody production (38).

## Possible Pathogenic Mechanisms of Neurological irAEs

Although the exact mechanism by which enhanced immune responses triggered by ICIs lead to the diverse spectrum of neurological irAE is not completely understood, there are multiple mechanisms suggested including potentiation of autoimmune paraneoplastic disorders or exacerbation of pre-existing autoimmunity that can be cancer-antigen driven or not (Figure 1).

Additionally, in cases of hypophysitis triggered by CTLA4 ICI treatment, CTLA4 expression by cells in the hypophysis and direct effect of the monoclonal antibodies and cytotoxicity has been suggested (43).

### Paraneoplastic Disorders Triggered by Immune Checkpoint Inhibitors

Paraneoplastic disorders result from an anti-tumor immune response against a shared autoantigen between the tumor (aberrant expression), and neural tissue (physiologic expression). Aberrant expression of a neural antigen by the tumor (in native or mutated forms), in conjunction with the enhanced T-cell responses after treatment with ICI may contribute to the development of a paraneoplastic syndrome, as onconeural-antigen-specific T-cells can cross the blood-brain barrier and cause neural-cell destruction (43).

An animal model of paraneoplastic cerebellar degeneration induced by treatment with CTLA4 ICI supports this hypothesis. In this model, mice were genetically engineered to express a shared antigen between their purkinje cells and cancer cells of an implanted tumor. When antigen specific lymphocytes were injected to the mice, they limited tumor growth slightly but had no neurological effects. When these were co-administered with a CTLA4 inhibitor, not only did that lead to tumor burden decrease but also to CD8+ T-cell mediated cytotoxicity on the Purkinje cells (44). A similar mechanism is suspected in patients with classic onconeural antibody positive paraneoplastic neurological disorders (45).

In cases of ICI triggered autoimmune encephalitis, CD4 and CD8 T-cells are involved. A study in a fatal case revealed robust and clonal CNS infiltration by CD4 and CD8 T-cells, whereas CD20 infiltrates were minimal. When compared to non-ICI-associated encephalitis tissue, there was predominance of activated memory phenotypes (46).

### Exacerbation of Pre-existing Autoimmunity

ICI treatment may unmask latent autoimmunity, in patients with underlying autoimmunity that remained subclinical before ICI treatment (43, 47). This was demonstrated in a case of a patient with radiologically isolated syndrome, who developed multiple sclerosis (MS) after ICI treatment. In this patient, CD4 and CD8 T-cell receptor repertoires were different in the CSF and tumor, indicating that ICI enhanced two pre-existing immune responses, one directed against the tumor, and another one directed against the CNS (48).

In a model of experimental autoimmune encephalitis (EAE), in PD-1 deficient mice, the disease course was accelerated compared to wild-type mice (49). In a model of EAE with anti-CTLA4 monoclonal antibody treated mice, disease severity was also exacerbated, indicating that CTLA4 pathway also plays a role attenuating T-cell activation in immune-mediated CNS demyelination (50).

Functional profiling of myelin-reactive CD4 T-cells in a patient who developed extensive demyelinating brain lesions after treatment with CTLA4 ICI, demonstrated high proliferation rates, pro-inflammatory cytokine production, and low production of the anti-inflammatory cytokine IL-10. This inflammatory CD4 T-cell phenotype was similar to that seen in patients with MS, and different than healthy controls (51).

## Clinical Approach Based on Neurological Presentation

### Central Nervous System Immune-Mediated Complications

#### Clinical Syndromes

##### Meningitis

Case vignette. A 60-year-old female was diagnosed with intestinal metastatic melanoma and underwent surgical resection of the intestinal tumor followed by treatment with nivolumab. After four treatments, she developed severe adrenal insufficiency and nivolumab was discontinued. Four months later, she presented to the emergency department with 3 days of severe headache, associated with nausea and vomiting. She also had short-term memory and word-finding difficulties, and gait impairment. On exam, she was somnolent, disoriented in time, she had mild expressive aphasia and gait ataxia. She was afebrile. MRI of the brain showed diffuse leptomeningeal enhancement. MRI of the cervical, thoracic and lumbar spine was unremarkable. CT scan of the chest, abdomen and pelvis showed decreased burden of lymphadenopathy, suggestive of positive response to nivolumab. CSF analysis showed lymphocytic pleocytosis (34 cells/mcL) and elevated protein (225 mg/dl). Extensive studies to rule out viral, bacterial and fungal infections were negative. CSF cytology was negative. Given suspected meningoencephalitis caused by nivolumab irAE, she received treatment with prednisone 60 mg daily for 1 week with significant clinical improvement and resolution of leptomeningeal enhancement on repeat MRI of the brain. Prednisone was tapered down by 10 mg per week and symptoms resolved.

The typical clinical presentation of meningitis is fever, headache, photophobia and neck stiffness. It may co-occur with encephalitis, as the case vignette above, and these patients also present with altered mental status. CSF may show pleocytosis and increased protein level (52). Brain MRI may show dural enhancement (53). Glial fibrillary acidic protein (GFAP) antibody may be found in these cases of meningitis but are often associated with encephalitis, myelitis or optic disk edema (30, 31). The reported incidence of meningitis in large studies of irAEs is below 1% (28, 29); however, this is likely an underestimation, as many cases are paucisymptomatic and are not investigated thoroughly (52).

Most patients with meningitis have favorable outcomes after ICI withdrawal and treatment with steroids, and ICI treatment resumption may be well-tolerated (52–54).

##### Encephalitis

Case vignette. A 52 year old female was diagnosed with stage IVb endometrial adenocarcinoma. She underwent hysterectomy with bilateral salpingo-oopherectomy and tumor debulking, followed by chemotherapy regimen consisting of carboplatin + paclataxel + dostralimab (PD-1 inhibitor, as part of research study). After completing 5 cycles of treatment, she developed subacute encephalopathy with hallucinations and tremor. MRI of the brain was unremarkable, and CSF analysis revealed positive N-methyl-D-aspartate receptor (NMDA-R) antibody. She was initially treated with 5 days of IV methylprednisolone 1,000 mg daily, followed by plasma exchange (6 treatments), with improvement of her symptoms, and was discharged to acute rehabilitation. She presented 2 weeks later with worsening encephalopathy, tremor and generalized weakness. Exam revealed nystagmus in all gaze directions, facial myoclonus, dysarthria, intention tremor of the upper extremities, myoclonus and appendicular ataxia. She quickly deteriorated requiring intubation. MRI was again unremarkable. Repeat CSF analysis showed lymphocytic pleocytosis (68 cells/mL), elevated protein (64 mg/dL) and positive oligoclonal bands. Infectious studies were negative. She had positive NMDA-R and GFAP antibodies in the CSF. She received 5 more days of IV methylprednisolone 1,000 mg daily and plasma exchange (5 treatments), with some improvement in her mental status. Repeat PET-CT revealed progression of metastasis. Further treatment for paraneoplastic encephalitis was cyclophosphamide and rituximab was discussed, however, given the progression of the underlying malignancy, decision was made for transition to palliative care. This is an example of a paraneoplastic autoimmune encephalitis with NMDA-R and GFAP antibodies in context of ICI treatment in a patient with endometrial adenocarcinoma.

Autoimmune encephalitis as a complication of ICI treatment can present in several forms. Presenting symptoms may include confusion, altered mental status, focal neurological deficits and seizures. Prompt recognition and treatment is essential as encephalitis may be a fatal complication (29, 55).

Limbic encephalitis is one of the most commonly reported CNS complications, presenting with altered mental status, anterograde amnesia, seizures and psychiatric symptoms. Brain MRI may show temporal lobe T2 hyperintensities (30, 31, 56–58). Neural antibodies such as antineuronal nuclear antibody type 1 (ANNA-1) and Ma2 can be found in cases of ICI related limbic encephalitis (30, 31, 57, 58). Unclassified neural-specific antibody case, and seronegative cases have also been described (30, 56). Additional cases of ICI related encephalitis associated with NMDA-R IgG have also been reported (30, 56).

Involvement not only of the limbic system, but also diencephalon and brainstem may occur in autoimmune encephalitis with Ma2 antibodies (33, 59–61). An increase in the incidence of Ma2 paraneoplastic syndromes has been noted since the introduction of ICI. The majority of these patients were middle-aged or elderly and had lung cancer or renal-cell carcinoma, whereas in the classic form of the disease, Ma2 is found in younger patients with seminoma. These patients also had less contrast enhancement on MRI that the classic Ma2 patients. Outcomes are poor, four of six patients died, and the other two had moderate to severe disability in this case series (33).

Cerebellar involvement can also occur, presenting sometimes as acute cerebellitis with abnormal T2 hyperintensities and contrast enhancement (62, 63), and in some cases with cerebellar atrophy (30, 31). Neural antibodies including ANNA-1, voltage-gated calcium channel (VGCC) and neural intermediate filament (NIF) have been reported in patients presenting with cerebellar ataxia (30). Involvement of the brainstem has also been reported (64), as well as involvement of the basal ganglia with hyperkinetic movement disorders and phosphodiesterase 10A (PDE10A) IgG (30, 65).

Autoimmune encephalitis is a severe complication of ICI treatment and patients may have poor prognosis, so treatment may require not only steroids but additional immunotherapy (such as intravenous immunoglobulin [IVIG], plasma exchange, rituximab or other), and permanent discontinuation of ICI (30, 31, 57, 58, 60, 64).

In some cases, posterior reversible encephalopathy syndrome (PRES) may also occur after ICI treatment (66–68). Presenting symptoms can include encephalopathy, vision loss, headache, seizures. MRI typically reveals T2 hyperintensities consistent with vasogenic edema. Concomitant PRES and peripheral nerve involvement was reported in a patient with leucine-rich glioma inactivated 1(LGI-1) IgG (30).

##### Myelitis

Involvement of the spinal cord may occur in isolation, or in combination with other syndromes (i.e. encephalomyelitis) (30). A case of longitudinally extensive transverse myelitis (LETM) with an excellent outcome after ICI discontinuation, steroids, IVIG and plasma exchange has been reported (69). A case of progressive myelopathy with collapsin response-mediator protein-5 (CRMP-5) antibodies experienced some improvement after ICI discontinuation, steroids and cyclophosphamide (70). One case of myelitis after radiation therapy and ICI has also been reported, with probable contributions of both therapies to the occurrence of this complication (71).

##### Other Inflammatory CNS Disorders

**Figure 2:**
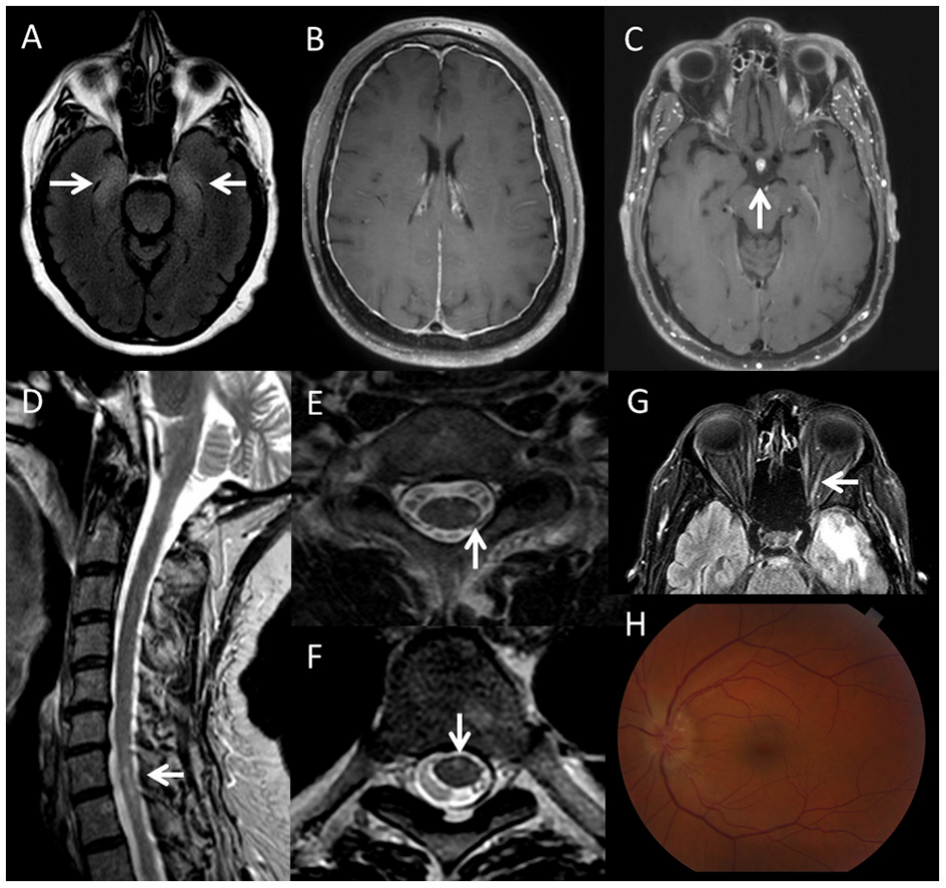
Imaging findings in patients with neurological immune related adverse events triggered by immune checkpoint inhibitors. **(A)** Axial brain MRI, fluid attenuation inversion recovery (FLAIR) sequence, showing bilateral T2-hyperintensity of the medial temporal lobes (right more than left), in a patient with limbic encephalitis associated with an unclassified neural-specific antibody, after treatment with nivolumab for melanoma. **(B)** Axial brain MRI, T1 post-gadolinium sequence showing dural enhancement in a patient with meningitis during treatment with nivolumab for sarcoma. **(C)** Axial brain MRI, T1 post-gadolinium sequence, showing enhancement of the hypophysis in a patient with hypophysitis after atezolizumab treatment. **(D)** Sagital T2 cervical spine MRI, **(E)** axial T2 cervical spine MRI and **(F)** axial T2 thoracic spine of a patient who developed short-segment (multiple sclerosis-like) spinal cord lesions (left lateral cord at C7–T1 and ventral cord at T5–T6) after treatment with nivolumab and ipilimumab for melanoma. **(G)** Axial FLAIR brain MRI showing T2 hyperintensity of the left optic nerve in the same patient. **(H)** Optic disc edema in a patient with bilateral optic neuritis triggered by atezolimumab treatment for small cell lung cancer, associated with positive CRMP-5 IgG.

Case vignette. A 57-year-old male was diagnosed with metastatic melanoma with a left temporal brain metastasis. After surgical resection and radiation, he underwent treatment with a combination of ipilimumab plus nivolumab. Two months after treatment initiation, he developed numbness of bilateral lower extremities, which progressed up to the mid abdominal region within 3 weeks. ICI treatment was discontinued with improvement of his sensory symptoms. Three months later, he developed a left eye monocular scotoma, which progressed to only light perception vision. An MRI of the brain and orbits showed gadolinium enhancement of the left optic nerve (Figure 2G) and the MRI of the spine showed a T2 hyperintense lesion involving the left hemicord at the level of C7-T1 (Figures 2D,E), and an additional lesion in the ventral cord at T5–T6 (Figure 2F). MOG-IgG and aquaporin-4-IgG were negative. CSF analysis showed normal cell count and protein. Oligoclonal bands were negative. He was treated with IV methylprednisolone, 1 g daily for three consecutive days, followed by an oral prednisone taper for suspected optic neuritis. His vision loss resolved. This is a representative case of CNS demyelination after ICI therapy. The patient's initial presentation was consistent with myelitis (not recognized at that time), and MRI of the spine showed short-segment lesions consistent with demyelination. This was followed by optic neuritis, with enhancement of the optic nerve.

CNS demyelination may occur after ICI therapy (30, 51). In patients with a history of MS, ICI therapy may trigger severe relapses (72, 73), and transition from radiologically isolated syndrome to clinically definite MS (48). In a study of 14 patients presenting with MS relapses during ICI therapy, 8 had a prior diagnosis of MS, 5 of whom experienced resolution of symptoms. Clinical presentation was severe in 3 patients, 2 of whom died. One patient was re-treated with ICI after presenting mild MS relapse, and his symptoms significantly worsened, with poor response to IV steroids. Of note, none of the patients continued their MS disease modifying therapy while on ICI treatment. Five patients received IV steroid treatment for MS relapse (73).

Two cases of neuromyelitis optica spectrum disorder (NMOSD) with positive aquaporin-4 IgG have been reported, presenting with LETM. An additional seronegative case of recurrent myelitis and optic neuritis (meeting NMOSD criteria) has been reported. The three patients experienced clinical improvement after treatment with IV steroids and plasma exchange (74–76).

Optic neuritis has also been reported in isolation, typically with bilateral optic nerve involvement. Optic disc edema and absence of pain seem to be common in ICI-related optic neuritis. Most patients had good outcomes after steroid treatment (77). Other neuro-ophthalmologic complications include uveitis, retinopathies, orbital inflammatory syndrome and Vogt-Koyanagi-Harada (77–79). Rare ocular paraneoplastic syndromes such as melanoma-associated retinopathy and bilateral diffuse uveal melanocytic proliferation have also been reported (30, 77).

Additional inflammatory CNS disorders described after ICI include CNS sarcoidosis (80), and intracranial vasculitis (81).

Hypophysitis may occur in up to 10% of patients on CTLA4 ICI therapy. Patients present with headache, fatigue, and anterior pituitary hormone deficiencies. Brain MRI may show enhancement and enlargement of the hypophysis (Figure 2). Treatment with steroids and hormone replacement are usually needed (43). ICI may be resumed after symptom resolution (54).

#### Diagnostic Findings in Patients With CNS Complications

MRI abnormalities are found in more than half of patients with CNS complications and they are consistent with the clinical syndromes (30). Brain MRI may reveal T2-hyperintensities in the mesial temporal lobes in patients presenting with limbic encephalitis (Figure 2). Additional signal abnormalities of the diencephalon and brainstem can be seen in patients with encephalitis associated with Ma2 antibody (33). Cerebellar atrophy can be observed in patients presenting with cerebellar ataxia (31). T2-hyperintensity of the basal ganglia can be seen with PDE-10A encephalitis (65). Abnormalities reported in patients presenting with meningoencephalitis include pachymeningitis, and diffuse perivascular enhancement in GFAP astrocytopathy cases (30, 31). In cases with optic nerve involvement, optic nerve T2-hyperintensity and contrast enhancement may be seen (77). Spinal cord MRI in patients presenting with myelitis may show short segment or longitudinally extensive lesions (69). Figure 2 shows some representative examples of imaging findings in patients with CNS complications after ICI.

In some patients with PET imaging of the brain, global hypometabolism and focal hypo- and/or hyper-metabolism has been found (30).

CSF abnormalities include lymphocytic pleocytosis and CSF restricted oligoclonal bands in up to 95% of the patients with a CNS presentation (23, 30, 31). EEG might also be helpful to evaluate for subclinical seizures (82).

#### Autoantibodies

Neural autoantibodies are more frequently found in patients presenting with CNS involvement, as opposed to peripheral nervous system (PNS) involvement (31). In a recent case series, more than half of the patients with CNS manifestations had neural-specific antibodies in serum, CSF or both. The majority were antibodies specific for intracellular targets, which are biomarkers of T-cell mediated cytotoxicity and some of them were yet molecularly unidentified (30).

As discussed above, Ma2 antibodies have been frequently found in patients with encephalitis and there seems to be an increased incidence in patients treated with ICI (31, 33, 53). Other antibodies reported include ANNA-1 (30, 31, 57, 58, 83, 84), glutamic acid decarboxylase (GAD65) (30, 85–87), NMDA-R (30, 56, 88), contactin-associated protein 2 (CASPR-2) (31, 89), GFAP (30, 31), LGI-1, CRMP5, amphiphysin, and SOX-1 (30). Novel antibodies recently described specific for PDE10A (19) and neural intermediate filament (NIF) (30), or molecularly undefined neural-restricted can also be found (30, 31).

Although many cases have neural specific antibodies, the absence of an antibody does not exclude the diagnosis of autoimmune encephalitis if the clinical presentation, timing and diagnostic testing is suggestive.

### Neuromuscular Immune-Mediated Complications

PNS complications may accompany CNS involvement or occur in isolation. They are two times more common than CNS irAEs.

#### Clinical Syndromes

##### Peripheral Nerve

Case vignette. A 52-year-old male with metastatic melanoma was treated with a combination of ipilimumab and nivolumab. After his fourth cycle, he developed back pain, and bilateral lower extremity numbness and weakness that progressed over 1 week. On exam, he had asymmetrical bilateral lower extremity weakness, predominantly distal, with areflexia and gait ataxia. The EMG showed evidence of an acute demyelinating sensory-motor polyneuropathy with conduction blocks. The MRI of the lumbar spine demonstrated subtle enhancement of the nerve roots of the cauda equina. The CSF had lymphocytic pleocytosis (23 cells/mcL) and elevated protein (159 mg/dL), with negative cytology. A PET-CT showed a favorable response of the melanoma to treatment. The patient received IV methylprednisolone 1 g daily for 5 days with improvement of his back pain. However, he had no significant change in his lower extremity weakness, and he developed a left facial palsy. He was then treated with IVIG 0.4 g/kg/day for 5 days. He also followed an oral prednisone taper. Three months after presentation, he had an almost complete recovery with only mild weakness of the left orbicularis oculi and left anterior tibialis. Unfortunately, his metastatic melanoma progressed, with development of new brain metastasis. He underwent whole-brain radiation and paclitaxel and carboplatin chemotherapy. After 1 month, patient transitioned to hospice care. This is a typical case of immune-mediated polyradiculoneuropathy triggered by ICI treatment.

Neuropathies are the most common peripheral nerve system complication (30). The clinical presentation is diverse, and sensory, motor and autonomic nerves may be affected. Cranial neuropathies and polyradiculoneuropathies are among the most common neuropathy presentations (90).

Excluding optic nerve involvement discussed in the CNS complications section, the most common cranial neuropathy affects the facial nerve, followed by the vestibulocochlear nerve. Bilateral involvement of these nerves is common (up to 44% of cases in a recent review) (91). Other reported cases include oculomotor, abducens, trigeminal, and glossopharyngeal involvement. Multiple cranial nerve involvement may also occur (27, 30, 53, 67, 77, 90, 91). Cranial neuropathies may present in conjunction with meningitis (90). Brain MRI can show enhancement of the affected nerves, and CSF may show pleocytosis and increased protein (91). Response to ICI discontinuation and steroid treatment is favorable in two thirds of the cases (91).

Polyradiculoneuropathies are typically subacute, although there are also acute cases with a Guillain-Barre like presentation (26, 30, 53, 67, 90, 92). The Miller-Fisher variant has also been reported (93). Polyradiculoneuropathies are more often demyelinating, but can also be axonal. CSF may show elevated protein level (albumin-cytological dissociation), and pleocytosis in some cases (90, 94).

Additional neuropathy phenotypes include neuralgic amyotrophy, phrenic neuropathies, mononeuritis multiplex, sensory neuronopathy, small fiber, or autonomic neuropathies (27, 30, 90).

Most neuropathies improve with ICI discontinuation and steroid treatment. Use of steroids has been associated with favorable outcomes, with improved disability scores (Inflammatory Neuropathy Cause and Treatment and modified Rankin Scale scores) (90). ICI re-challenge can be considered in less severe cases (54). Even if Guillain-Barre-like syndrome is classically treated with IVIG, when related to ICI, steroids are the first-line of treatment (95).

##### Neuromuscular Junction

Case vignette. A 73 year old male was diagnosed with skin melanoma stage IIIA (lymph node involvement). After surgical resection of melanoma, he initiated adjuvant therapy with pembrolizumab. The patient had a history of ocular myasthenia gravis (MG) diagnosed 3 years prior, when he presented with diplopia and had positive muscle acetylcholine receptor (AChR) antibody. He had no evidence of thymoma. He was stable on 10 mg of prednisone daily. Two weeks after the first pembrolizumab dose, the patient developed diplopia, generalized weakness, dyspnea and dysphagia that progressed rapidly. He presented to the Emergency Department and required intubation due to respiratory failure. He was diagnosed with MG crisis and received treatment with plasma exchange, IVIG and increased dose of prednisone. After hospitalization for 1 month, he had some clinical improvement and was eventually discharged to a long term care facility with a tracheostomy and percutaneous endoscopic gastrostomy tube. He continued oral prednisone 60 mg and IVIG monthly.

The patient presented to our institution 10 months later for a second opinion due to persistence of diplopia, weakness, dysphagia and dyspnea. On exam, he had fatigable weakness of the upper and lower extremities, neck flexion weakness, flaccid dysarthria, mild facial weakness and diplopia. The EMG showed a diffuse disorder of neuromuscular transmission with a superimposed myopathy with diaphragmatic involvement. AChR binding antibody (0.31 nmol/L, normal <0.02), AChR modulating antibody (61%, normal <20), and striational antibody (1:15360, normal <1:120) were positive. Troponin was elevated (60 ng/L, normal <15) and CK was normal. A left deltoid muscle biopsy showed chronic myopathic changes. An echocardiogram showed normal ejection fraction. Rituximab was added to his regimen for MG treatment and beta-blockers were started for his cardiomyopathy.

This is a representative case of an exacerbation of pre-existing myasthenia gravis with ICI, but also a superimposed myopathy and cardiomyopathy triggered by the ICI treatment, which were missed at the time of initial presentation.

Flare up of pre-existing MG and new onset MG after ICI treatment has been reported. ICI-related MG compared to spontaneous MG, is characterized by a more severe clinical presentation, with acute onset and rapid deterioration. Patients with ICI-related MG more frequently develop facial muscle weakness, bulbar symptoms and respiratory involvement. For this reason, many patients require mechanical ventilator support (96, 97). ICI-related MG seems to have a higher fatality rate than other irAEs, and it tends to occur earlier after ICI initiation (median 29 days for MG vs. 61–80 days for other events) (28).

Some patients develop MG overlapping with myositis, with elevated creatine kinase (CPK), and electrodiagnostic features of both MG and myositis (96, 97). They may also develop myocarditis, which may present as heart failure or arrhythmias, or subclinical elevation of serum cardiac troponin T and reduction of ejection fraction in echocardiogram (96). Myositis and myocarditis has been associated with worse outcomes and higher mortality (28). However, some of the cases initially diagnosed as MG based on the finding of positive AChR antibodies, did not have electrodiagnostic features of MG, and had elevated CPK (97), therefore, it is possible that some of these patients had in fact myositis.

Ptosis and diplopia in isolation due to neuromuscular junction involvement has been reported rarely (77).

Discontinuation of ICI and steroids might not be enough in severe MG cases, and treatment with IVIG and plasma exchange is often necessary (97). Rituximab or eculizumab can also be considered.

Lambert-Eaton myasthenic syndrome associated with P/Q voltage gated calcium antibodies has also been reported in two patients with SCLC and squamous-cell lung cancer. Both of them presented with proximal weakness, and one also had ptosis. Nerve conduction studies showed characteristic facilitation of the compound muscle action potential with exercise. ICI was discontinued and treatment included cholinesterase inhibitors, 3,4-diaminopyridine and steroids, with improvement of symptoms. One of the patients was subsequently rechallenged with ICI and had worsening of weakness (98, 99).

##### Muscle

Case vignette. A 68 year old female with metastatic melanoma was treated with nivolumab. After the second dose of nivolumab, she developed generalized myalgias. Nivolumab was continued for 2 more doses, with myalgias getting progressively worse. After the fourth dose, the patient had developed weakness of her upper and lower extremities, dysphagia, dysarthria, and dyspnea. On exam, she was found to have proximal bilateral symmetric weakness of the upper and lower extremities (deltoid, biceps, triceps, hip flexors and quadriceps). She also had weakness of the neck flexors and flaccid dysarthria. CPK was above 4,000 IU/L. Striational antibody was positive (1:61440, normal <1:120). Needle EMG was consistent with a proximal myopathy, showing short duration and low-amplitude motor unit potentials, with early recruitment. Fibrillation potentials and myotonic discharges were also observed, indicating the presence of necrosis, splitting, or vacuolization of muscle fibers. Repetitive stimulation (2-hertz) showed no decrement. Biopsy of the left deltoid was consistent with a necrotizing myopathy. She was treated with IV methylprednisolone 1,000 mg IV for 5 days, followed by oral prednisone 60 mg daily with gradual improvement of her symptoms. She was on prednisone 60 mg daily for 2 months, followed by a slow taper over 4 months. Six months after presentation, her neurological exam was normal.

This is a representative case of myositis after ICI treatment with a good outcome. Patients typically present with prominent oculo-bulbar and proximal limb muscles involvement (30, 100). Myalgia is a frequent symptom, and usually precedes muscle weakness (92, 101). Respiratory muscle involvement has been reported in up to 42% of cases, often requiring intubation (100). The majority of patients present a rapid progression of symptom severity. CPK is usually significantly elevated above 1,000 IU (median 680-2,668 U/L) (63, 100–102). EMG shows myopathic features (30, 100, 101).

The clinical presentation of ICI related myositis may resemble MG given the significant ocular and bulbar involvement, but patients do not typically report fluctuation of weakness (100). Additionally some cases have muscle AChR-IgG, and this may lead to a misdiagnosis of MG, but the electrophysiological findings (no decrement), and absence of response to acetylcholinesterase inhibitors are not consistent with MG (100, 101, 103).

Of note, concomitant myocarditis may occur in up to 40% of the patients (102). Myocarditis is a life-threatening complication and therefore it should be investigated in all patients presenting with myositis and/or myasthenia gravis (102, 104).

Myositis typically improves after ICI withdrawal and treatment with steroids, but unfavorable outcomes are reported, with 20% mortality in a recent series (30, 100, 101). Patients with myocarditis and/or MG overlap have longer hospitalizations, more frequently require additional immunosuppression, and are more likely to experience respiratory failure and death, compared to patients with myositis alone (102). Respiratory failure requiring intubation is also a poor prognosis factor (100).

#### Diagnostic Findings

In peripheral nerve cases, electrophysiologic findings may provide supportive findings for an immune-related neuropathy, such as a non-length-dependent, axonal, or demyelinating polyradiculoneuropathy, sensory neuronopathy, or mononeuritis multiplex (90). This is helpful to differentiate from chemotherapy induced neuropathy, which typically presents with a length-dependent distal symmetric sensory neuropathy.

MRI of the brain and spine may demonstrate nerve root gadolinium enhancement in cases with cranial neuropathies and polyradiculoneuropathies (90). Nerve biopsy may be considered in these cases with nerve root enhancement to differentiate an inflammatory response from metastatic infiltration or when a vasculitic neuropathy is suspected (90). CSF analysis may show albuminocytological dissociation in inflammatory polyradiculoneuropathies (23).

In cases of myositis, EMG shows myopathic pattern, predominantly in the proximal limbs (30, 100, 101, 103). EMG may also identify subclinical axial and limb involvement in patients with oculobulbar presentation without limb weakness (100). Repetitive nerve stimulation showing more than 10% decrement, and single-fiber EMG showing increased jitter, might be helpful for cases with myasthenia gravis (95). CPK is elevated in the majority of patients with myopathy, and aldolase may also be elevated. CPK and aldolase levels are not as high as other immune mediated necrotizing myopathies (100). Muscle MRI may reveal intramuscular T2-hyperintensity with or without contrast enhancement (100, 102). Abnormalities in orbit MRI with T2-hyperintensity and enlargement of extraocular muscles have also been observed in patients with myositis and ocular involvement. PET may show increased FDG uptake in the muscles (100). Muscle biopsy frequently shows multifocal clusters of necrotic fibers, which seems to be a distinctive pattern of ICI-related myositis (30, 100, 101). Inflammation with predominantly lymphocytic T-cell infiltration has also been described (101, 102). Cardiac muscle may also be affected, with elevated troponin in 62–78% of the patients (30, 101), and decreased ejection fraction in echocardiography in about one third of the patients (30).

#### Autoantibodies

Muscle AChR antibodies may be present in serum of patients presenting with MG but they may also be found in patients with myositis and do not establish a diagnosis of MG. It has been hypothesized that the presence of AChR-IgG is a biomarker of the underlying malignancy or can be related to antigen release from muscle destruction (101, 105). The presence of muscle AChR-IgG in patients with thymoma treated with ICI predicted the development of myositis. Striational antibody is positive in up to 47% of patients presenting with myositis, often at high titer (100).

## When to Suspect a Neurologic irAE and What to Do

New onset neurological symptoms in patients treated with ICIs that are not easily explained by metabolic abnormalities or metastatic disease should prompt consideration for an autoimmune complication, especially if the patient presented within 3 months from ICI initiation or escalation/change.

Evaluation of patients on treatment with ICI presenting with neurological symptoms starts with a thorough neurological history and examination. Subacute onset of symptoms is a clue to inflammatory etiology. The localization of the symptoms should guide further investigations.

In most cases with CNS involvement, MRI of the brain and/or MRI of the spine will help to exclude a structural pathology related to the tumor such as brain metastasis or spinal cord compression as the cause of the patient's symptoms. MRI and CSF analysis can also help evaluate for carcinomatous meningitis, which can be challenging to diagnose. Additionally, CSF analysis in patients presenting with meningitis and encephalitis should include infectious studies to exclude bacterial, viral and fungal meningitis in immunocompromised patients (82). Evaluation of metabolic abnormalities in patients presenting with encephalopathy should also be pursued. Neural antibody evaluation should be obtained in serum and CSF, given that the finding of a specific neural antibody supports an immune related adverse event.

In peripheral neuropathies, EMG and nerve conduction studies are important for differential diagnosis mainly with chemotherapy induced neuropathies.

In a myositis presentation, EMG, CPK levels, myositis antibody panel (including anti-SRP,-hydroxymethylglutaryl-CoA reductase) should be obtained. If the presentation suggests MG, EMG should include repetitive nerve stimulation and SFEMG and antibody evaluation should also include muscle AChR and MUSK specificities. Muscle biopsy can be considered. It is critical to test troponin and obtain an echocardiogram in all patients presenting with myositis to rule out cardiac involvement.

Given the severity of neurological immune adverse events and the risk for disabling deficits if the diagnosis and treatment are delayed, involvement of a neurologist with experience in these diseases in a multidisciplinary team is of great importance. Diagnostic delay may lead to continuation of ICI therapy and worse outcomes.

## General Recommendations for Treatment

The mainstay of treatment of neurological irAEs is to withhold the ICI. Due to the long pharmacokinetic and pharmacodynamic effects of these drugs, immunosuppression (usually steroids) to reduce the immune response is often needed. Guidelines for the treatment of irAEs according to the organ involved and the toxicity grade have been issued by the American Society of Clinical Oncology (82), the Society for Immunotherapy of Cancer Toxicity Management Working Group (21), and more recently the National Comprehensive Cancer Network (NCCN) (54).

### Acute Treatment

ICI discontinuation is usually the first step of treatment, although it is noteworthy that neurological improvement has been reported in mild and moderate cases receiving acute immunosuppressive therapy even without ICI discontinuation (30).

Corticosteroids are the first-line of acute treatment in patients with severe neurological irAE, with either IV methylprednisolone or oral prednisone (27, 95). Our approach is to use IV methylprednisolone 1,000 mg daily for 5 days, followed by 1,000 mg weekly for 6–12 weeks in patients with CNS involvement (except for mild meningitis). In patients with neuromuscular complications, prednisone 1 mg/kg daily can also be considered (35).

In severe cases, ICI discontinuation and steroid treatment might not be sufficient. IVIG at 0.4 g/kg/day for 3 consecutive days, followed by weekly for 6–12 weeks, or plasma exchange every other day for 5–7 treatments can be used (27, 35). There is also some evidence that patients with ICI-related MG may benefit from upfront IVIG or plasmapheresis, regardless of initial severity (97).

In refractory cases, second-line treatments including rituximab or cyclophosphamide (intravenous or oral) can be considered. For patients with pre-existing paraneoplastic neurological syndromes and with severe CNS manifestations, prompt initiation of these agents may be appropriate (35). The type of neural antibodies found may guide selection of treatment. In patients with neural antibodies directed against cell surface antigens such as NMDA-R-IgG, the antibodies are pathogenic and B-cell depleting therapies like rituximab are more commonly used, whereas in patients with neural antibodies directed against intracellular antigens such as ANNA-1-IgG, the process is mediated by cytotoxic T-cells and therefore are preferably treated with cyclophosphamide.

Natalizumab has also been reported to be beneficial in steroid refractory paraneoplastic encephalitis after ICI treatment but metastatic brain disease should be a consideration in patients with malignancies prone to metastasize to the brain. It has been suggested that natalizumab, unlike other immunosuppressants, has the potential of reducing adhesion and migration of leukocytes through the blood-brain barrier without affecting the systemic immune reaction against malignancy (84).

Alemtuzumab (antiCD52 monoclonal antibody) and abatacept (CTLA-4 agonist) have been used successfully to treat steroid refractory myositis and myocarditis (106, 107).

Infliximab (anti TNF-alpha inhibitor) has also been used, especially in patients with co-occurring non-neurological irAE (27, 108). Of note, a recent study showed decreased survival in ICI treated melanoma patients who received anti-TNF agents for the treatment of irAEs (109).

### Long-Term Treatment

In general, prolonged immunosuppression is not recommended, as it may have a deleterious effect on cancer outcome. Maintaining steroids for at least 3 months might be appropriate given the long half-life of ICI (3–4 weeks) (23). Concern was raised about concomitant use of steroids or other immunosuppressant during ICI treatment may affect the efficacy of ICI, as in one study in patients with advanced NSCLC treated with PD-L1, baseline use of prednisone >10 mg daily was associated with poorer outcome (110). However, in patients with melanoma treated with nivolumab and ipilimumab, steroids did not reduce ICI efficacy (111–113).

In case of relapses, or severe cases with prolonged recovery, maintenance immunosuppression can be considered on a case to case basis (35). In our and others experience, agents used for long-term therapy include azathioprine up to 2–3 mg/Kg/day, mycophenolate mofetil 2,000–3,000 mg/day, and rituximab 1,000 mg day 0 and 14 with repetition every 6 months as well as cyclophosphamide for 6 months (oral 0.8–2 mg/Kg/day based on renal function, or intravenous 0.6–1.0 g/m^2^ monthly) (27, 35).

### ICI Re-challenge

The safety of ICI reintroduction after the development of a neurological irAE is unknown and the decision should be made on a case to case basis. Re-challenge might be considered in cases of advanced malignancies with limited treatment options that have responded well to neurological irAE treatment or only had low severity irAEs. In the latter cases, even though rare for neurological irAEs ICIs can also be continued. Overall, the reported incidence of irAE recurrence or development of a new irAE after ICI re-challenge is 39–55% (114–116). Although previous guidelines recommended avoiding ICI re-challenge after CNS autoimmunity (21, 117), it might be well-tolerated in patients with less severe CNS complications (31, 54). In patients with myositis alone (without concomitant myocarditis), ICI rechallenge may be reasonable after resolution of myositis. In a recent case series, one of five patients with myositis rechallenged on ICI had a subsequent flare (102), whereas in another series all three patients rechallenged developed serious complications (two recurrent myositis and one colitis) (100). In general, ICI rechallenge in patients with severe acute manifestations is not recommended (54).

In a recent case series of patients with severe (grade ≥ 3) CNS and PNS neurological irAE, half of the 10 patients re-challenged had a relapse within 6 months of the initial events, and most of them had not received immunosuppression to treat the first event. The authors propose that immunosuppression could reduce this rate of recurrence. They also recommend observation for 2–8 weeks after resolution or stabilization of neurological symptoms prior to considering ICI reinitiation (27).

It remains to be elucidated whether addition of immunosuppression to ICI rechallenge could reduce the risk of relapse.

### ICI Treatment in Patients With Pre-existing Autoimmunity and Paraneoplastic Disorders

Pre-existing autoimmune disorders can be exacerbated by treatment with ICI. Case series have reported flares of underlying autoimmune disease in 29–55% of patients. These include psoriasis, rheumatoid arthritis, thyroid disease, and neurological disorders such as multiple sclerosis and myasthenia gravis (30, 118–121). In an observational study among more than 4,438 patients receiving ICIs, pre-existing autoimmune disease was associated with an increase in hospitalizations with irAE diagnoses and steroid treatment (122).

In general, patients with preexisting autoimmune disease which is non-life threatening, and well-controlled with low level or no immunosuppression, could be considered for ICI treatment (113). Clinical evidence of prior CNS inflammatory disorders (such as multiple sclerosis) is not considered a formal contraindication to treatment with ICI in a life-threatening malignancy. However, the risk benefit ratio should be evaluated on a case by case basis (44).

A recent study showed that 3 out of 5 patients who developed autoimmune encephalitis after treatment with atezolizumab had the HLA-B^*^27:05 genotype, which has been associated with inflammatory conditions such as ankylosing spondylitis, psoriasis and inflammatory bowel disease. This suggests that HLA-B^*^27:05 genotype could potentially be a risk factor that needs further investigation (123).

Pre-existing paraneoplastic syndromes (not only neurological, but also others such as hypertrophic osteopathy and dermatomyositis), can be worsened by ICI treatment in up to half of the patients (124). Patients with thymoma and pre-existing AChR autoantibodies had an increased risk of developing myositis after treatment with avelumab (105). In a recent case series, three patients with pre-existing paraneoplastic syndromes affecting the CNS and positive neural antibodies (P/Q-type-VGCC, ANNA1, and CRMP-5 IgG) presented severe worsening of their neurological symptoms, which did not improve after immunosuppressive therapy (30). Several additional cases with worsening of pre-existing paraneoplastic neurological syndromes, mostly associated with ANNA-1-IgG, and one case with coexisting NMDA-R IgG have been reported in the literature. The outcomes of these patients were poor, with high mortality (half of the cases) (57, 58, 83, 84, 125, 126). Neural antibodies have also been detected in patients with paraneoplastic syndromes triggered by ICI, before initiation of treatment, when they were asymptomatic (33). Caution should be exercised when using ICIs in patients with pre-existing paraneoplastic neurological syndromes and neural antibodies. Although the cost-benefit ratio of universal neural antibody screening prior to initiation of ICI treatment is unclear, it could be considered in patients with malignancies classically associated with neurological autoimmunity such as SCLC and thymoma (127, 128).

## Outcome

In our recent Mayo Clinic case series, neurological outcomes were unfavorable (residual severity ≥3) in one third of the patients, and favorable (severity grade 1) in 33% (30). This is similar to other series that reported that approximately one third of the patients were left with residual deficits after development of a neurological irAE (24).

Outcomes are worse in patients with CNS involvement compared to PNS involvement, except for those patients presenting with meningoencephalitis, who have better outcomes (31). Other factors associated with unfavorable outcomes include older age, female gender, lung cancer, higher severity at onset, and absence of preceding or accompanying non-neurological autoimmunity (30).

Severe clinical presentation and poor outcomes despite aggressive treatment are observed in patients who had pre-existing paraneoplastic CNS syndromes and positive classic paraneoplastic neural-specific antibodies prior to ICI initiation, and therefore ICI should be used with caution in these patients (30). The clinical benefit and cost-effectivity of neural antibody screening prior to ICI initiation is still unclear, but it has been proposed for patients with malignancies classically associated to paraneoplastic syndromes, such as SCLC and thymoma (30).

Use of steroids to treat severe neurological irAE has been associated with favorable outcomes (27, 90). In patients with MG, treatment with IVIG or plasmapheresis as additional first-line therapy, may also lead to better outcomes than steroids alone (97).

In rare cases, relapses may occur after discontinuation of immunosuppressive therapy (30). Risk for relapses may be higher among patients with involvement of both the CNS and PNS (compared to involvement of CNS or PNS in isolation). Risk of relapse is also higher in patients retreated with ICI (27).

Fatality rates are higher in patients presenting with MG, especially when in combination with myositis and myocarditis (28); and in patients with encephalitis, with up to 50% mortality in a series of patients with Ma2 limbic encephalitis (31).

## Conclusion

Immune checkpoint inhibitors have revolutionized cancer treatment and its use is expected to grow. Clinicians must be aware of the diverse spectrum of neurological toxicities associated with cancer immunotherapy. An increase in the incidence of paraneoplastic syndromes is likely to occur with the widespread use of immune checkpoint inhibitors for the treatment of various malignancies, especially if used in those malignancies most frequently associated with spontaneous paraneoplastic syndromes such as SCLC, gynecological cancers and thymic tumors. Although most neurologic irAE can be managed by discontinuation of the ICI and steroid treatment, some cases may be severe and life-threatening. Early recognition and treatment is critical to maximize recovery. Multidisciplinary approach including oncology and autoimmune neurology, as well as other specialists depending on other systems affected (cardiology in patients with myocarditis) is crucial to improve patient's outcomes. This is an ongoing need as novel oncological treatments may lead to neurological complications not only related to ICI, but also chimeric antigen receptor (CAR) T-cell therapy, which has been associated with unique neurological complications known as immune effector cell-associated neurotoxicity syndrome (129).

## Author Contributions

CV-S drafted and edited the manuscript. AZ critical review and editing the manuscript.

## Conflict of Interest

AZ has a patent on PDE10A-IgG as a marker of neurological autoimmunity. The remaining author declares that the research was conducted in the absence of any commercial or financial relationships that could be construed as a potential conflict of interest.

## Abbreviations

AChR: acetylcholine receptor
ANNA-1: antineuronal nuclear antibody type 1
CASPR-2: contactin-associated protein 2
CNS: central nervous system
CPK: creatin kinase
CRMP-5: collapsin response-mediator protein-5
CSF: cerebrospinal fluid
CTLA-4: cytotoxic T lymphocyte associated antigen 4
EEG: electroencephalogram
EMG: electromyogram
GAD65: glutamic acid decarboxylase isoform 65
GFAP: glial fibrillary acidic protein
ICI: immune checkpoint inhibitors
IVIG: intravenous immunoglobulin
irAE: immune related adverse events
LGI-1: leucine-rich glioma inactivated 1
MG: myasthenia gravis
MRI: magnetic resonance imaging
NIF: neural intermediate filament
NMDA-R: N-methyl-D-aspartate receptor
NSCLC: Non-small cell lung cancer
PD-1: programmed death-1 receptor
PD-L1: programmed death-1 receptor ligand
PDE10A: phosphodiesterase 10A
PNS: peripheral nervous system
SCLC: small cell lung cancer
TCR: T-cell receptor.
